# Plasma Cyclic Guanosine Monophosphate Is a Promising Biomarker of Clinically Significant Portal Hypertension in Patients With Liver Cirrhosis

**DOI:** 10.3389/fmed.2021.803119

**Published:** 2022-01-04

**Authors:** Lukas Sturm, Dominik Bettinger, Lisa Roth, Katharina Zoldan, Laura Stolz, Chiara Gahm, Jan Patrick Huber, Marlene Reincke, Rafael Kaeser, Tobias Boettler, Wolfgang Kreisel, Robert Thimme, Michael Schultheiss

**Affiliations:** ^1^Department of Medicine II, Medical Center University of Freiburg, Faculty of Medicine, University of Freiburg, Freiburg, Germany; ^2^Berta-Ottenstein-Program, Faculty of Medicine, University of Freiburg, Freiburg, Germany; ^3^IMM-PACT-Program, Faculty of Medicine, University of Freiburg, Freiburg, Germany

**Keywords:** cyclic guanosine monophosphate, liver cirrhosis, portal hypertension, varices, transjugular intrahepatic portosystemic shunt (TIPS)

## Abstract

**Introduction:** Despite intensive research, reliable blood-derived parameters to detect clinically significant portal hypertension (CSPH) in patients with cirrhosis are lacking. As altered homeostasis of cyclic guanosine monophosphate (cGMP), the central mediator of vasodilatation, is an essential factor in the pathogenesis of portal hypertension, the aim of our study was to evaluate plasma cGMP as potential biomarker of cirrhotic portal hypertension.

**Methods:** Plasma cGMP was analyzed in cirrhotic patients with CSPH (ascites, *n* = 39; esophageal varices, *n* = 31), cirrhotic patients without CSPH (*n* = 21), patients with chronic liver disease without cirrhosis (*n* = 11) and healthy controls (*n* = 8). cGMP was evaluated as predictor of CSPH using logistic regression models. Further, the effect of transjugular intrahepatic portosystemic shunt (TIPS) placement on plasma cGMP was investigated in a subgroup of cirrhotic patients (*n* = 13).

**Results:** Plasma cGMP was significantly elevated in cirrhotic patients with CSPH compared to cirrhotic patients without CSPH [78.1 (67.6–89.2) pmol/ml vs. 39.1 (35.0–45.3) pmol/l, *p* < 0.001]. Of note, this effect was consistent in the subgroup of patients with esophageal varices detected at screening endoscopy who had no prior manifestations of portal hypertension (*p* < 0.001). Cirrhotic patients without CSPH displayed no significant elevation of plasma cGMP compared to patients without cirrhosis (*p* = 0.347) and healthy controls (*p* = 0.200). Regression analyses confirmed that cGMP was an independent predictor of CSPH (OR 1.042, 95% CI 1.008–1.078, *p* = 0.016). Interestingly, portal decompression by TIPS implantation did not lead to normalization of plasma cGMP levels (*p* = 0.101).

**Conclusions:** Plasma cGMP is a promising biomarker of CSPH in patients with cirrhosis, especially with respect to screening for esophageal varices. The lacking normalization of plasma cGMP after portal decompression suggests that elevated plasma cGMP in cirrhotic portal hypertension is mainly a correlate of systemic and splanchnic vasodilatation, as these alterations have been shown to persist after TIPS implantation.

## Introduction

The development of clinically significant portal hypertension (CSPH) is a milestone in the disease progression of liver cirrhosis as it underlies numerous complications such as variceal bleeding, ascites and hepatorenal syndrome and is associated with significantly reduced survival (1). Accordingly, diagnosis of CSPH is of great prognostic relevance. The gold standard for the diagnosis of portal hypertension is invasive hepatic venous pressure gradient (HVPG) measurement (2). Despite intensive research on alternative, non-invasive tools to detect CSPH, no reliable blood-derived parameters or scoring systems for this purpose could be implemented into clinical care so far (3). Several studies in the animal model have demonstrated that altered homeostasis of cyclic guanosine monophosphate (cGMP), the central mediator of vasodilatation, is a substantial pathomechanism of cirrhotic portal hypertension: While intrahepatic cGMP activity is decreased, cGMP activity is increased in extrahepatic blood vessels, contributing to the state of sinusoidal constriction and systemic and splanchnic vasodilatation pathognomonic for advanced liver cirrhosis (4–7). These data suggest that altered plasma cGMP levels could be an indicator of the presence of portal hypertension in patients with cirrhosis. Therefore, the aim of our study was to investigate alterations of plasma cGMP in different stages of chronic liver disease and to evaluate cGMP as a potential biomarker of CSPH.

## Materials and Methods

### Patient Selection

In total, 110 participants were enrolled in the study. This included 70 cirrhotic patients with CSPH: 39 patients had ascites and 31 patients had esophageal varices. All varices patients were free of ascites at the time of study inclusion and in the past. Twelve of the 31 patients had a history of variceal bleeding, while in 19 patients varices were not previously known, but detected during screening endoscopy (with no bleeding at the time of diagnosis). Further, 21 cirrhotic patients without CSPH, 11 patients with chronic liver disease without liver fibrosis or cirrhosis and eight healthy controls were included. Patients were recruited during in- or out-patient treatment at the University Medical Center Freiburg, Germany, between 06/2017 and 12/2019.

Diagnosis of liver cirrhosis was based on distinct sonographic, clinical and laboratory findings. Liver function was assessed using the Child-Pugh score and the Model of End Stage Liver Disease (MELD). In patients with CSPH, the presence of ascites was confirmed by sonography and the presence of clinically relevant varices according to the Baveno VI consensus definition (medium or large varices requiring treatment by non-selective betablockers or endoscopic band ligation) was assessed by endoscopy (8). In cirrhotic patients without CSPH, the absence of varices and ascites was verified by means of endoscopy and sonography and the medical records were reviewed to exclude a history of varices or ascites. In patients with chronic liver disease without liver fibrosis this was confirmed by sonography and transient elastography (liver stiffness <6.5 kPa). Apart from chronic liver disease, patients had no other severe cardiovascular, respiratory, renal or metabolic conditions.

### Assessment of CGMP Levels

Venous blood samples were obtained from all participants at the time of study inclusion. Blood samples were centrifuged immediately and plasma was stored at −80°C until cGMP measurement. In 13 patients with cirrhosis, additional blood samples were obtained between one and 12 months after implantation of a transjugular intrahepatic portosystemic shunt (TIPS) to study the effects of portal decompression.

CGMP levels were determined in plasma using an enzyme-linked immunosorbent assay (ELISA) by Research & Diagnostic Systems Inc., MN, US (KGE003). Sample preparation and conduction of the assay were performed according to the manufacturer's specifications.

### Patients' Consent and Ethics Approval

All patients gave written informed consent to their participation. The study was approved by the local ethics committee of the University of Freiburg, Germany, (no. EK 85/19) and is in accordance with the Declaration of Helsinki.

### Statistical Analyses

The study was a comprehensive analysis of plasma cGMP levels of patients in different stages of chronic liver disease and portal hypertension. Categorical variables are expressed as absolute and relative frequencies, continuous variables as median with interquartile range. In the absence of a Gaussian distribution of the data, differences between patient groups were assessed by Chi square tests for categorical variables and by Mann Whitney U, Wilcoxon rank sum or Kruskal Wallis tests, as applicable, for continuous variables. Predictors of CSPH were evaluated by fitting uni- and multivariable logistic regression models. Demographic data, etiology of liver disease and the MELD score as measure of liver function were included in the models. Further, the Lok index and the aspartate-aminotransferase to platelet ratio index (APRI) as fibrosis scores were included, as they showed good performance in the detection of CSPH previously (9). Due to the limited number of patients, the scores were entered into multivariable regression separately in order minimize bias by interactions. Further, the platelet count/spleen diameter (PC/SD) ratio was included (10). A *p* value of < 0.05 was considered significant.

## Results

### Patient Characteristics

Table 1 summarizes patient characteristics. Cirrhotic patients with and without CSPH were of comparable age and gender distribution with a median age of 60 (55–67) and 61 (54–67) years (*p* = 0.925) and a majority of 49 (70.0%) and 15 (71.4%) males, respectively (*p* = 0.900). As to be expected, patients with CSPH had more advanced liver disease, highlighted by a MELD score of 11 (8–14) in comparison to patients without CSPH with a MELD score of 7 (7–8; *p* = 0.007). Alcoholic liver disease was the leading etiology in patients with CSPH (*n* = 45, 64.3 %), while cirrhotic patients without CSPH mostly had viral liver disease (*n* = 15, 71.4 %).

**Table 1:** Patient characteristics.

	**Cirrhosis with CSPH (n** **=** **70)**		**Cirrhosis no CSPH (*n* = 21)**	**No cirrhosis (*n* = 11)**	**Controls (*n* = 8)**
	**Ascites (*n* = 39)**	**Varices (*n* = 31)**			
Age [years]	60 (56–72)	60 (55–66)	61 (54–67)	47 (41–63)	48 (42–58)
Sex					
Male	28 (71.8)	21 (67.7)	15 (71.4)	7 (63.6)	4 (50.0)
Female	11 (28.2)	10 (32.3)	6 (28.6)	4 (36.4)	4 (50.0)
Etiology					
Viral	6 (15.4)	6 (19.4)	15 (71.4)	8 (72.7)	
Alcoholic	29 (74.4)	16 (51.6)	1 (4.8)		
Other	4 (10.2)	9 (29.0)	5 (23.8)	3 (27.3)	
Child-Pugh					
A	4 (10.3)	20 (64.5)	18 (85.7)		
B	26 (66.7)	9 (29.0)	3 (14.3)		
C	9 (23.1)	2 (6.5)	0		
MELD	11 (8–13)	10 (8–14)	7 (7–8)		
Platelets [10^∧^3/μl]	118 (83–161)	84 (54–119)	124 (98–190)	238 (221–270)	
INR	1.2 (1.1–1.2)	1.2 (1.1–1.3)	1.0 (1.0–1.1)	1.0 (0.9–1.0)	
Bilirubin [mg/dl]	1.0 (0.7–2.2)	1.3 (0.9–2.3)	0.6 (0.4–1.0)	0.5 (0.4–0.7)	
Albumin [g/dl]	3.1 (2.8–3.3)	4.2 (3.6–4.3)	4.6 (4.2–4.8)	4.6 (4.4–4.7)	
Creatinine [mg/dl]	1.2 (0.9–1.7)	0.8 (0.7–1.1)	0.9 (0.8–1.1)	1.0 (0.8–1.1)	
AST (U/l)	47 (36–72)	46 (34–63)	46 (30–58)	27 (22–39)	
ALT (U/l)	24 (20–35)	35 (28–42)	43 (28–63)	41 (25–85)	
Spleen diameter [mm]	130 (120–160)	150 (130–180)	125 (110–145)	110 (90–120)	
Lok index	1.51 (0.98–2.51)	2.11 (1.31–2.59)	0.63 (−0.42–1.07)	−1.63 (−2.21–1.20)	
APRI	0.71 (0.46–1.13)	1.26 (0.58–2.15)	0.53 (0.42–1.08)	0.23 (0.16–0.33)	
PC/SD ratio	804 (626–1.309)	590 (376–1.032)	946 (718–1.736)	2,164 (2046–2.700)	

*APRI, aspartate-aminotransferase to platelet ratio index; ALT, alanine-aminotransferase; AST, aspartate-aminotransferase; CSPH, clinically significant portal hypertension; INR, international normalized ratio; MELD, Model of End Stage Liver Disease; PC/SD ratio, platelet count/spleen diameter ratio*.

### Elevated Plasma cGMP Levels in Patients With Clinically Significant Portal Hypertension

**Figure 1:**
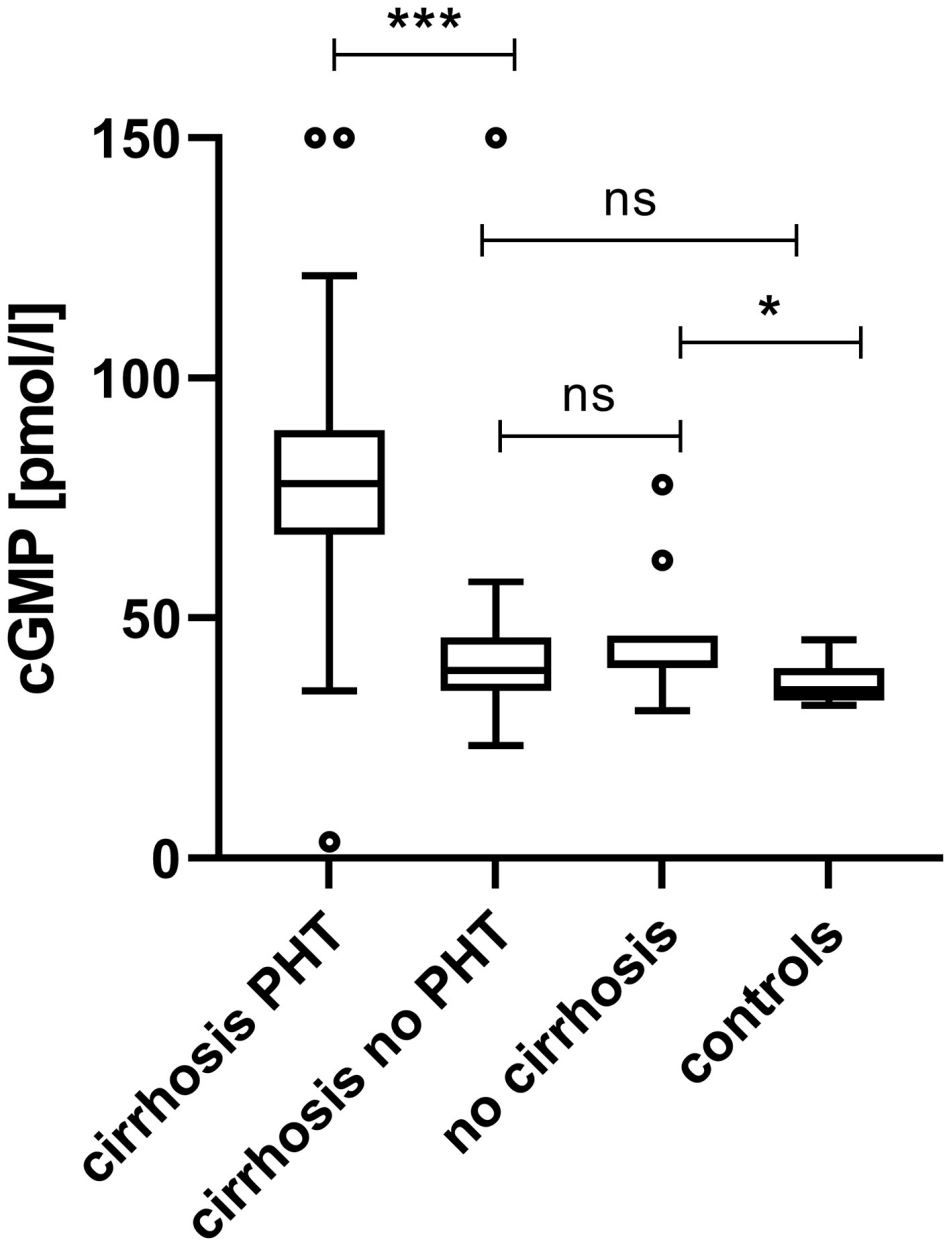
Plasma cGMP in patients in different clinical states of chronic liver disease and clinically significant portal hypertension. Plasma cGMP was significantly elevated in cirrhotic patients with CSPH in comparison to cirrhotic patients without CSPH [78.1 (67.6–89.16) pmol/ml vs. 39.1 (35.0–45.3) pmol/l, *p* < 0.001]. There was no significant difference in cGMP levels between cirrhotic patients without CSPH compared to patients with chronic liver disease without liver cirrhosis [40.3 (39.7–46.3) pmol/l, *p* = 0.347] or healthy controls [35.0 (32.9–39.1) pmol/l, *p* = 0.200]. For better visualization cGMP measurements of two patients with CSPH (320.1 pmol/ml and 249.6 pmol/l) and one cirrhotic patient without CSPH (249.2 pmol/l) are plotted at 150 pmol/l. ***p < 0.001; *p < 0.05; ns, not significant.

**Figure 2:**
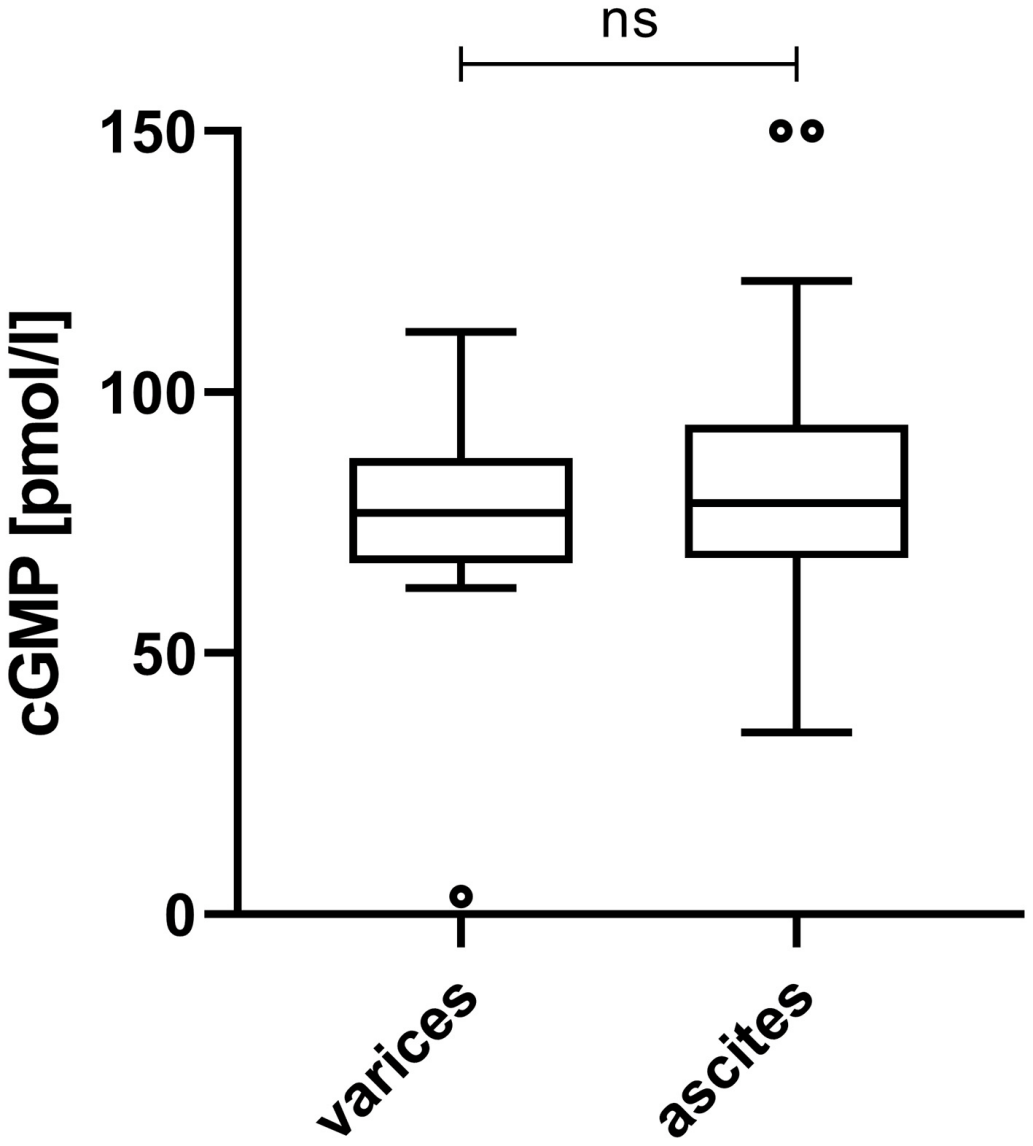
Plasma cGMP in cirrhotic patients with varices and ascites. Plasma cGMP levels were independent of the manifestation of portal hypertension, as there was no significant difference between varices patients with varices and patients with ascites [76.9 (70.3–93.1) pmol/ml vs. 78.9 (69.1–93.0) pmol/l, *p* = 0.537]. For better visualization cGMP measurements of two ascites patients (320.1 pmol/ml and 249.6 pmol/l) are plotted at 150 pmol/l. ns, not significant.

**Figure 3:**
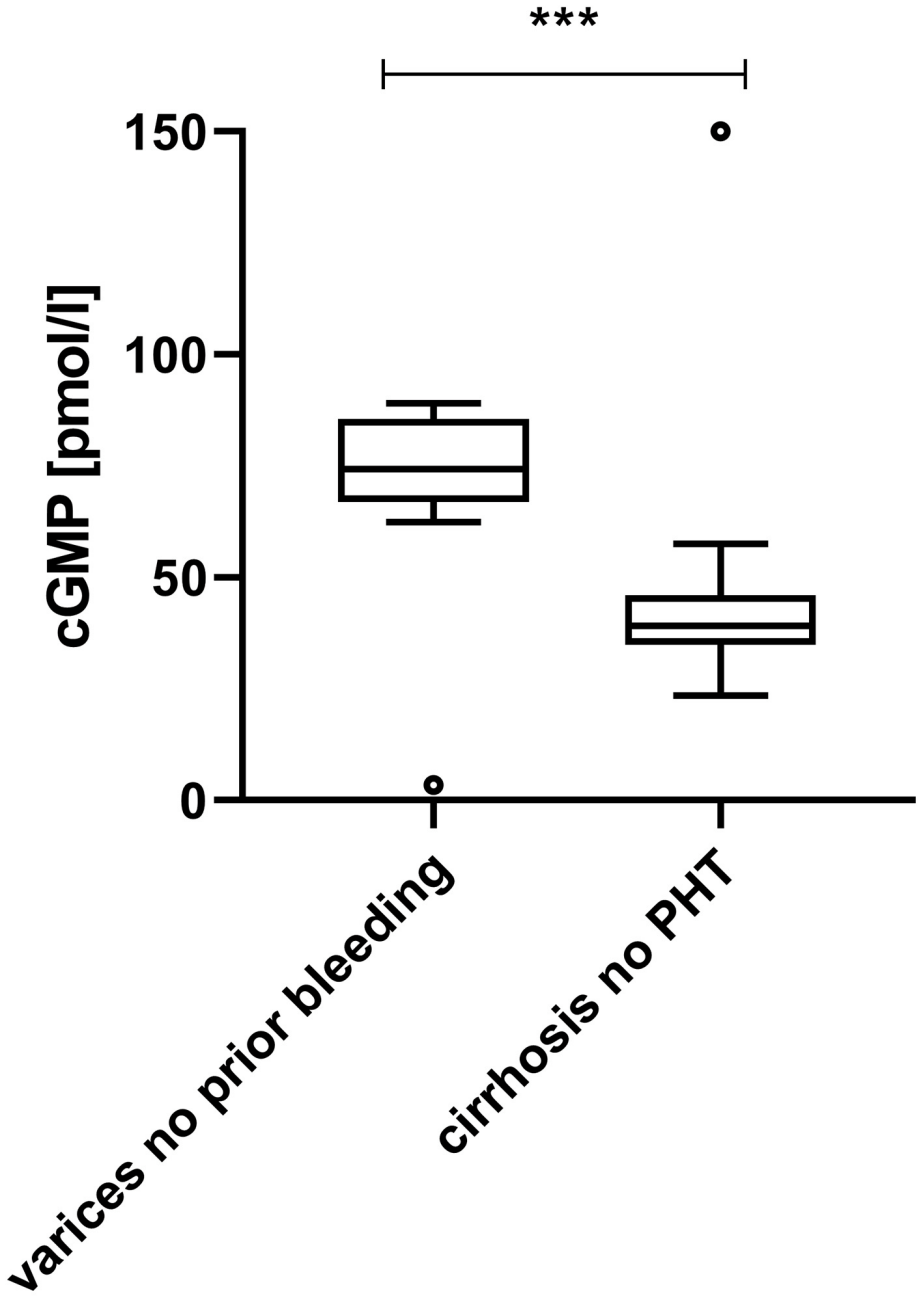
Plasma cGMP in patients with varices diagnosed at screening endoscopy in comparison to cirrhotic patients without clinically significant portal hypertension. Patients with esophageal varices detected by screening endoscopy without other manifestations of portal hypertension displayed significantly elevated plasma cGMP in comparison to cirrhotic patients without CSPH [74.3 (67.0–85.0) pmol/l vs. 39.1 (35.0–45.3) pmol/l, *p* < 0.001]. For better visualization the cGMP measurement of one patient without CSPH (249.2 pmol/l) is plotted at 150 pmol/l. ***p < 0.001.

Plasma cGMP was significantly elevated in cirrhotic patients with CSPH in comparison to cirrhotic patients without CSPH [78.1 (67.6–89.2) pmol/ml vs. 39.1 (35.0–45.3) pmol/l, *p* < 0.001]; Figure 1. There was no significant difference in cGMP levels between cirrhotic patients without CSPH compared to patients with chronic liver disease without liver cirrhosis [40.3 (39.7–46.3) pmol/l, *p* = 0.347] or healthy controls [35.0 (32.9–39.1) pmol/l, *p* = 0.200]. The elevation in plasma cGMP was independent of the manifestation of portal hypertension, as there was no significant difference between patients with varices and patients with ascites [76.9 (70.3–93.1) pmol/ml vs. 78.9 (69.1–93.0) pmol/l, p = 0.537]; Figure 2. Of note, the subgroup of patients with esophageal varices detected during screening endoscopy without other manifestations of portal hypertension also displayed significantly elevated plasma cGMP [74.3 (67.0–85.0) pmol/l] in comparison to cirrhotic patients without CSPH (*p* < 0.001); Figure 3. Comparison of etiologies of liver disease among patients with CSPH revealed no significant difference in plasma cGMP between patients with alcoholic liver disease [76.8 (69.1–87.7 pmol/l)], viral liver disease [75.5 (61.6–87.0 pmol/l)] and other etiologies [87.5 (71.8–97.5) pmol/l], *p* = 0.246.

### Evaluation of Plasma cGMP as Predictor of Clinically Significant Portal Hypertension

To explore the predictive effect of plasma cGMP for the presence of CSPH and to adjust for differences between patient groups, potential predictors of portal hypertension were included in a regression model. Multivariable regression demonstrated that plasma cGMP indeed was an independent predictor of CSPH (OR 1.042, 95% CI 1.008–1.078, *p* = 0.016), besides viral liver disease (OR 0.032, 95% CI 0.003–0.415, *p* = 0.008) and the Lok index (OR 1.650, 95% CI 1.006–2.708, *p* = 0.047); Table 2.

**Table 2:** Logistic regression models of predictors of clinically significant portal hypertension.

	**Univariable regression**			**Multivariable regression**		
**Parameters**	**OR**	**95% CI**	***p*-value**	**OR**	**95% CI**	***p*-value**
Male gender	1.256	0.550–2.872	0.588			
Age	1.043	1.006–1.081	0.022			
Viral liver disease	0.153	0.063–0.370	<0.001	0.032	0.003–0.415	0.008
MELD	1.166	0.977–1.392	0.089			
cGMP	1.076	1.047–1.106	<0.001	1.151	1.058–1.252	0.001
PC/SD ratio	0.999	0.998–0.999	<0.001			
Lok index	1.706	1.230–2.366	0.001	1.650	1.006–2.708	0.047
APRI	2.842	1.202–6.716	0.017			

*APRI, aspartate-aminotransferase to platelet ratio index; cGMP, cyclic guanosine monophosphate; MELD, Model of End Stage Liver Disease; PC/SD ratio, platelet count/spleen diameter ratio*.

### Effects of Non-selective Beta Blocker Treatment and Transjugular Intrahepatic Portosystemic Shunt Placement on Plasma cGMP

Of the 12 included patients with a history of variceal bleeding, seven patients (58.3%) received treatment with non-selective beta blockers (NSBBs) for secondary prophylaxis of variceal hemorrhage. Comparison with the five patients (41.7%) without NSBB treatment showed no significant difference in plasma cGMP between the groups [84.7 (65.1–87.5) pmol/l vs. 85.1 (75.9–87.1), *p* = 0.999].

**Figure 4:**
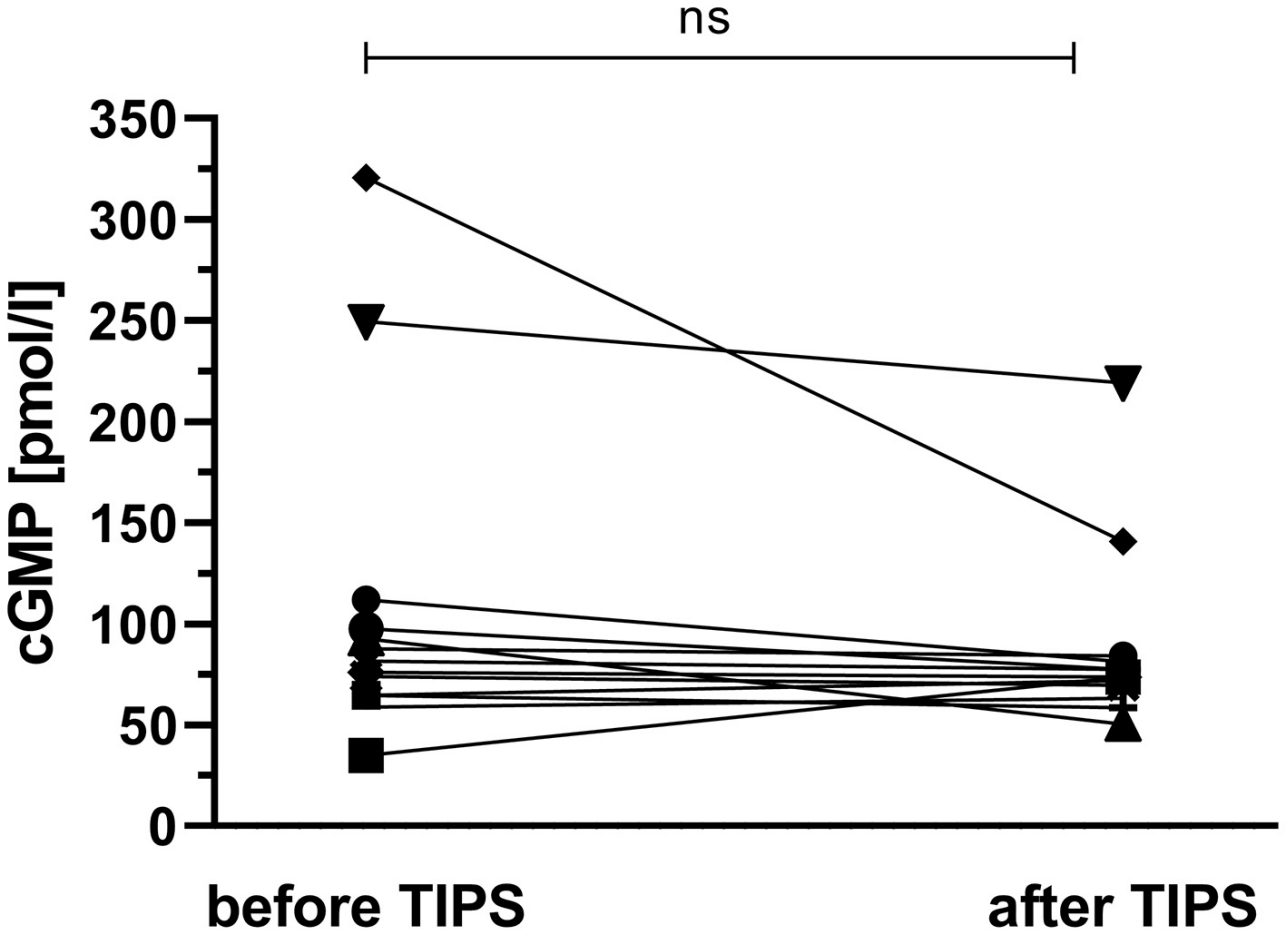
Effect of transjugular intrahepatic portosystemic shunt placement on plasma cGMP. Following TIPS implantation, plasma cGMP showed a decrease in 10 out of 13 patients (76.9 %). However, the intra-individual changes in cGMP levels did not reach significance (*p* = 0.101). ns, not significant.

The effect of portal decompression on plasma cGMP was studied in 13 cirrhotic patients who underwent TIPS implantation. The patients' pre-TIPS portosystemic pressure gradient (PSG) was 20 (19–28) mmHg. Graphical exploration revealed no unequivocal link of plasma cGMP to pre-TIPS PSG measurements; Supplementary Figure 1. TIPS placement reduced the patients' PSG to 11 (10–12) mmHg. Following TIPS implantation, plasma cGMP showed a decrease in 10 out of 13 patients (76.9 %); Figure 4. However, the intra-individual changes in cGMP levels were not significant (*p* = 0.101).

## Discussion

Besides fibrotic re-modeling of the liver tissue, impaired vasotonus regulation is the most important factor in the pathogenesis of portal hypertension in liver cirrhosis (11). Various studies in the animal model of portal hypertension have demonstrated decreased hepatic cGMP activity with reflectively increased splanchnic and systemic cGMP activity (4–7). These alterations are believed to be a major contributing factor to the state of profuse hepatic vascular resistance and hyperdynamic splanchnic and systemic circulation that characterizes cirrhotic portal hypertension (12, 13). This pathophysiological background suggests that cGMP could be a biomarker of portal hypertension. As the diagnosis of CSPH in patients with cirrhosis is of great prognostic relevance, means to detect and monitor CSPH foregoing the invasive gold standard of HVPG measurement are subject to intensive research. First, a variety of promising instrument-based parameters such as transient elastography or magnetic resonance imaging have been investigated in this context (14–16). Second, different blood-derived parameters and scoring systems have been evaluated (9, 17). In comparison to instrument-based methods, a broad availability and uncomplicated conduction may be considered potential benefits of blood-derived tests. However, no reliable blood-derived parameters could be incorporated into clinical routine so far (3). Against this background, we set out to investigate alterations of plasma cGMP in chronic liver disease with special focus on evaluating its potential as a biomarker of CSPH.

Indeed, we observed significantly increased plasma cGMP in patients with cirrhosis who had CSPH compared to cirrhotic patients without CSPH (*p* < 0.001). Logistic regression analyses adjusting for factors such as liver function confirmed that plasma cGMP was an independent predictor of CSPH in the patient collective investigated. These results are in conformity with previous reports of elevated plasma cGMP in patients with cirrhosis and CSPH in smaller patient collectives (18–21). In contrast to previous studies, we systematically investigated patients in different clinical states of portal hypertension. Here, we observed that plasma cGMP was not linked to the manifestation of portal hypertension, as there was no significant difference between patients with varices and patients with ascites. Further sub-analyses revealed that the group of patients with varices detected during screening endoscopy and no prior manifestations of portal hypertension also displayed significantly elevated cGMP levels in comparison to cirrhotic patients without portal hypertension (*p* < 0.001). Naturally, prediction of CSPH by non-invasive markers is most relevant in this early stadium of portal hypertension in which the condition has not yet been unmasked by variceal hemorrhage or the development of ascites. Hence, this finding suggests that cGMP could be a valuable parameter in screening for esophageal varices. Another important aspect of our study is that we also incorporated patients with chronic liver disease without liver fibrosis and healthy controls. Notably, their plasma cGMP levels were not significantly different from those of cirrhotic patients without portal hypertension. This finding supports the conclusion that elevated plasma cGMP is indeed primarily related to the development of portal hypertension and not to liver cirrhosis alone. Further, we studied the effects of TIPS placement on plasma cGMP in a subset of cirrhotic patients. Here, we observed no significant decrease in plasma cGMP after TIPS implantation. Prior studies have shown that while the portosystemic shunt offers effective portal decompression, it does not resolve the state of systemic vasodilatation characteristic of cirrhotic portal hypertension (22–24). Considering this aspect, the persistent elevation of plasma cGMP after TIPS insertion suggests that altered plasma cGMP in cirrhotic portal hypertension is mainly a correlate of systemic vasodilatation. In any case, this finding argues against a usefulness of cGMP for monitoring the absence or recurrence of portal hypertension after TIPS implantation on a pathophysiological basis. However, future studies in larger patient collectives should investigate the relation between response of plasma cGMP and clinical response following TIPS placement. Furthermore, the effect of treatment with NSBBs on plasma cGMP was studied in patients with a history of variceal bleeding. Comparison of patients who received NSBBs for secondary prophylaxis of variceal hemorrhage to patients without a NSBB medication revealed no significant difference in plasma cGMP between the patient groups. Importantly, in patients with NSBB treatment no plasma cGMP measurements prior to commencement of NSBB therapy were available as reference in the present study, so an impact of NSBBs on plasma cGMP cannot be excluded on the basis of the present results. Another aspect that needs to be addressed is the impact of co-morbidities on plasma cGMP. For example, elevated plasma cGMP levels have been described in patients with congestive heart failure (25). To minimize bias regarding this aspect we only included patients who had no severe internistic co-morbidities. However, future studies will have to consider the influence of co-morbidities when evaluating the specificity of elevated plasma cGMP for the prediction of CSPH in patients with cirrhosis.

Our study has some limitations that need to be discussed: We incorporated 110 patients and controls in our analysis, which was a sufficiently high number to detect significant differences in plasma cGMP between patients in different stages of chronic liver disease and portal hypertension. Still, it is important to keep in mind that our results are derived from a limited number of patients in each subgroup. Another limitation of our study are inhomogeneities between patient groups. This aspect showed especially with respect to etiology of liver disease: While alcoholic liver disease was the leading etiology in patients with CSPH, patients without CSPH mostly had viral liver disease. To adjust for this fact, we applied logistic regression models. Importantly, plasma cGMP prevailed as independent predictor of CSPH in multivariable regression. Still, further studies in larger, more homogeneous patient collectives are necessary to confirm the findings of the present study and to systematically investigate if plasma cGMP levels are affected by different etiologies of liver disease.

In conclusion, the present study demonstrates that plasma cGMP homeostasis is significantly altered in cirrhotic patients with CSPH. Our results propose that cGMP could serve as a blood-derived biomarker of CSPH, especially with respect to screening for esophageal varices. Follow-up studies are necessary to evaluate plasma cGMP's diagnostic performance in the prediction of CSPH in comparison to other non-invasive parameters and scoring systems.

## Data Availability Statement

The raw data supporting the conclusions of this article will be made available by the authors, without undue reservation.

## Ethics Statement

The studies involving human participants were reviewed and approved by Ethics Committee University of Freiburg, Germany. The patients/participants provided their written informed consent to participate in this study.

## Author Contributions

LStu, DB, and MS: study concept, design, and drafting of the manuscript. LStu, DB, LR, KZ, LSto, CG, and MS: acquisition of data. LStu, LR, DB, and MS: analyses and interpretation of data. TB, JH, MR, RK, WK, and RT: critical revision of the manuscript for important intellectual content. MS: supervision. All authors contributed to the article and approved the submitted version.

## Funding

LStu is supported by the Berta-Ottenstein-Program, Faculty of Medicine, University of Freiburg, Germany. RK is supported by the IMM-PACT-Program, Faculty of Medicine, University of Freiburg, Germany. The article processing charge was funded by the Baden-Wuerttemberg Ministry of Science, Research and Art and the University of Freiburg in the funding programme Open Access Publishing.

## Conflict of Interest

The authors declare that the research was conducted in the absence of any commercial or financial relationships that could be construed as a potential conflict of interest.

## Publisher's Note

All claims expressed in this article are solely those of the authors and do not necessarily represent those of their affiliated organizations, or those of the publisher, the editors and the reviewers. Any product that may be evaluated in this article, or claim that may be made by its manufacturer, is not guaranteed or endorsed by the publisher.

## Supplementary Material

Supplementary Figure 1 Plasma cGMP and portosystemic pressure gradient. The graph shows the relation of plasma cGMP levels and portosystemic pressure gradient (PSG) measurements in 13 cirrhotic patients with implantation of a transjugular intrahepatic portosystemic shunt (TIPS). For better visualization the cGMP measurement of two patients with very high plasma cGMP measurements (249.6 pmol/l and 320.1 pmol/l) are plotted at 150 pmol/l.

## Abbreviations

APRI: aspartate-aminotransferase to platelet ratio index
cGMP: cyclic guanosine monophosphate
CSPH: clinically significant portal hypertension
HVPG: hepatic venous pressure gradient
MELD: Model of End Stage Liver Disease
PC/SD: platelet count/spleen diameter
PSG: portosystemic pressure gradient
TIPS: transjugular intrahepatic portosystemic shunt.
