# Supplementary figures and images for: Plasma Cyclic Guanosine Monophosphate Is a Promising Biomarker of Clinically Significant Portal Hypertension in Patients With Liver Cirrhosis
Source: Front Med (Lausanne). 2022 Jan 4;8:803119. doi: 10.3389/fmed.2021.803119 (PMC8764357; doi:10.3389/fmed.2021.803119)

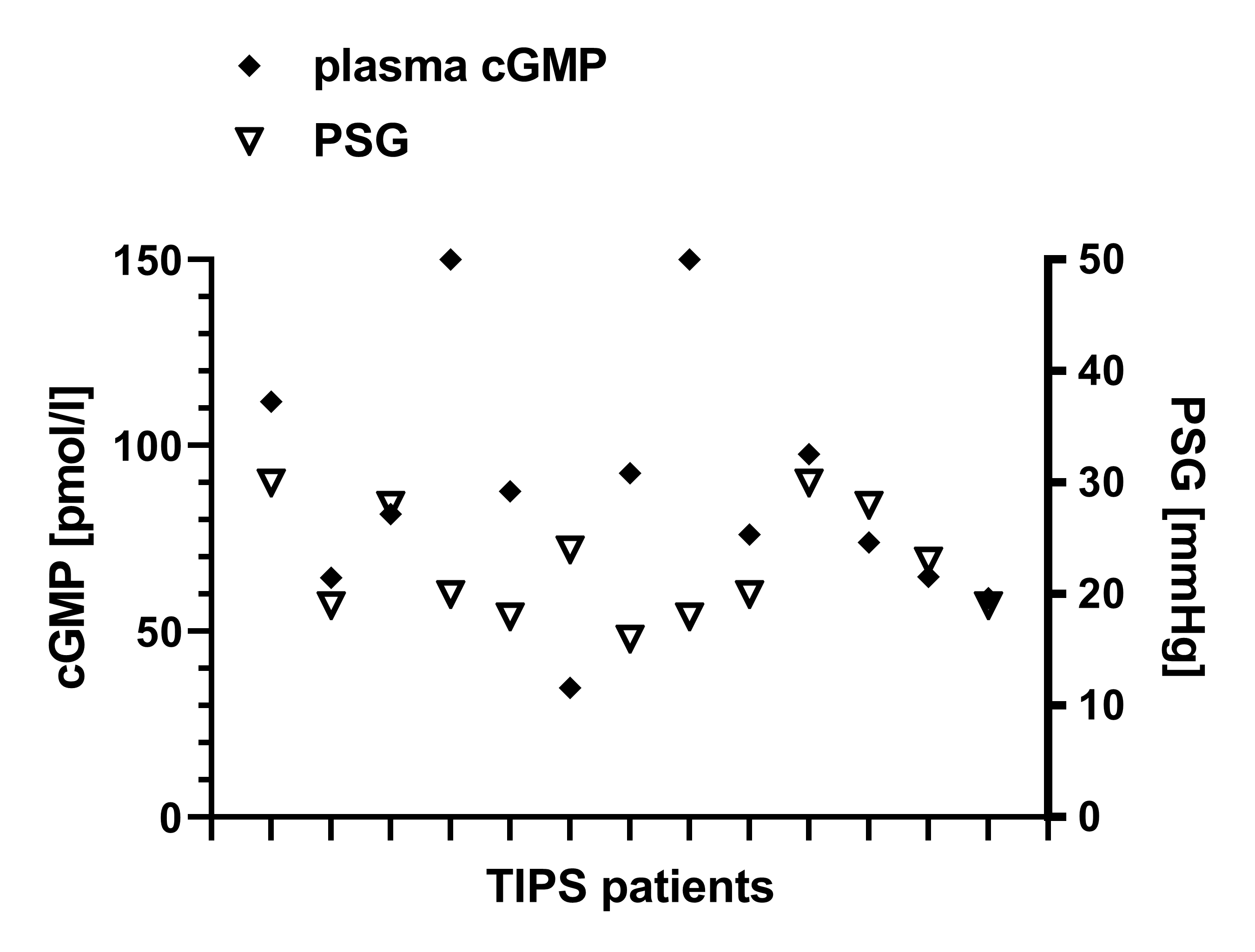

Supplement: Supplementary Figure 1 — Plasma cGMP and portosystemic pressure gradient. The graph shows the relation of plasma cGMP levels and portosystemic pressure gradient (PSG) measurements in 13 cirrhotic patients with implantation of a transjugular intrahepatic portosystemic shunt (TIPS). For better visualization the cGMP measurement of two patients with very high plasma cGMP measurements (249.6 pmol/l and 320.1 pmol/l) are plotted at 150 pmol/l. [file Image_1.tif]
